# Human herpesvirus 4 and adaptive immunity in Alzheimer’s disease

**DOI:** 10.1038/s41392-020-0125-y

**Published:** 2020-04-13

**Authors:** Jian-Sheng Kang, Pei-Pei Liu

**Affiliations:** Clinical Systems Biology Laboratories, The First Affiliated Hospital of Zhengzhou University, Zhengzhou, 450052 Henan China

**Keywords:** Diseases of the nervous system, Neurodevelopmental disorders

In a recent paper published in *Nature*, Gate et al. reported adaptive immune changes in Alzheimer’s disease (AD), discovered CD8^+^ T effector memory CD45RA^+^ (T_EMRA_) cells with proinflammatory and cytotoxic functions in AD patients, and identified two T_EMRA_ cell-recognized antigens of human herpesvirus 4 (HHV-4), also known as Epstein–Barr virus (EBV).^1^ The findings suggested that adaptive immunity might have a role in AD progression.

In this article, the authors performed comprehensive analyses of four cohorts to explore the adaptive immunity in AD. In the first cohort, using mass cytometry to study the peripheral blood mononuclear cells (PBMCs) of patients with mild cognitive impairment (MCI) or AD, the authors detected an increased population of CD3^+^CD8^+^CD27^−^CD45RA^+^ T_EMRA_ cells. In the second cohort, a negative correlation was revealed between T_EMRA_ cells and cognition in MCI and AD patients. By stimulating PBMCs with phorbol 12-myristate 13-acetate and ionomycin, T_EMRA_ cells from patients with MCI or AD demonstrated potent effector functions, such as secreting proinflammatory cytokines, including interferon-γ (IFN-γ) and tumor necrosis factor (TNF), which was consistent with the upregulated T cell receptor (TCR) and cytokine signaling in T_EMRA_ cells. In the third cohort, the authors detected the increased localization of T_EMRA_ cells adjacent to amyloid-β (Aβ) plaques in the leptomeninges adjacent to the hippocampus and in the cerebrospinal fluid (CSF) of patients with AD. In the fourth cohort, the authors revealed the cytotoxic function of clonal T_EMRA_ cells from the CSF of patients with AD, including high expression levels of cytotoxic effectors (granule protein 7, NKG7 and granzymes A, GZMA) and the pro-aging factor (beta-2-microglobulin, B2M). Therefore, the authors demonstrated the proinflammatory (IFN-γ and TNF) and cytotoxic functions (NKG7, GZMA and B2M) of T_EMRA_ cells from patients with AD. Since T effector memory cells are associated with immunological memory, the authors further determined the antigens that caused specific clonal expansion of T_EMRA_ cells in AD, including *Herpesviridae* EB nuclear antigen 3 (EBNA3A) and the EBV/HHV-4 trans-activator protein BZLF1.

Unlike the prevalence rates of herpes simplex virus types 1 (HSV1/HHV-1) and 2 (HSV2/HHV-2) that vary markedly by countries and population subgroups,^2^ EBV/HHV-4 infects more than 95% of people within the first decades of life in all populations.^3^ However, only 6% of AD brains were EBV/HHV-4 positive, while 45% of peripheral blood leukocyte samples from AD patients were positive for EBV/HHV-4.^4^ As cautioned by the authors, there is no suggestion for a causal link between EBV/HHV-4 infectivity and the onset of AD. Interestingly, all EBV/HHV-4-positive brains were from the type 4 allele of the apolipoprotein E gene (*APOE ε4*) carriers.^4^ The *APOE ε4* allele accounted for 70% of the population attributable risk for AD,^5^ and the molecular pathobiology of *APOE ε4* remains elusive.^6^ A significant association between HHV positivity and AD was found only in patients who had been diagnosed with AD for 6.6 years or more, especially in female patients.^7^ APOE ε4 might facilitate the entry of HHV into the brain more efficiently than APOE ε3.^8^ Gate et al. did not report the type of *APOE* allele of AD patients or the length of duration that patients had AD. If these links could be confirmed, the work by Gate et al. might potentially and partially provide an explanation for the late aggressive AD progression of *APOE ε4* carriers. Previous studies about HHV and AD were largely inconsistent and lacked of solid mechanisms and robust associations with a specific viral species, as we recently reviewed.^9^ Consequently, Tony Wyss-Coray and his colleagues made an overall important and reliable step in the field.

**Fig. 1:**
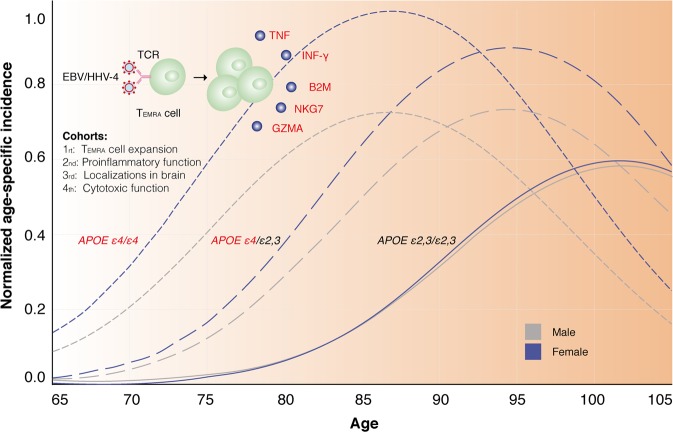
Four cohorts demonstrated the proinflammatory and cytotoxic functions of CD8^+^ TEMRA cells stimulated by EBV/HHV-4. The predicted AD prevalence among individuals with zero, one or two copies of the *APOE ε4* allele^5^ was also shown for the potential association between *APOE ε4* and HHV. APOE ε4 can facilitate the entry of HHV into the brain.^8^ Fonts highlighted in red represent harmful factors. IFN-γ and TNF secreted by CD8^+^ T_EMRA_ cells are proinflammatory factors, and NKG7, GZMA, and B2M released by CD8^+^ T_EMRA_ cells are cytotoxic or pro-aging factors

Gate et al.^1^ unambiguously demonstrated the proinflammatory and cytotoxic functions of T_EMRA_ cells stimulated by EBV/HHV-4 antigens (Fig. 1) as a part of adaptive immunity in AD patients. On the other hand, the fibrilization of Aβ can be stimulated by HHV as a protective innate immune response.^10^ Overall, the interactions among Aβ, APOE ε4 and HHV and the crosstalk between innate and adaptive immunity deserve further elegant efforts. Considering the frequency of the *APOE ε4* allele among the human population (~14%) and late-onset familial AD (~40%),^6,9^ *APOE ε4* carriers with AD are worthy of consideration for special therapeutic diagnoses and interventions as a specific subtype of familial AD,^6^ such as the influence of the adaptive immunity evoked by HHV.
